# Supplementary material for: Improvement of Qualitative Analyses of Aliphatic Alcohols Using Direct Catalytic Fuel Cell and Chemometric Analysis Format
Source: Sensors (Basel). 2024 May 18;24(10):3209. doi: 10.3390/s24103209 (PMC11124824; doi:10.3390/s24103209)
Supplement: Supplementary file 1 [file sensors-24-03209-s001.zip › SM1.pdf]

SM1: Supplementary Materials of the paper:

IMPROVEMENT OF QUALITATIVE ANALYSES OF ALIPHATIC ALCOHOLS, USING CATALYTIC FUEL CELL AND CHEMOMETRIC ANALYSIS FORMAT.

Mauro Tomassetti[1]*, Federico Marini[1]*, Riccardo Pezzilli[2], Mauro Castrucci[1], Corrado Di Natale[3], Luigi Campanella[1]

[1]Department of Chemistry, University of Rome, “La Sapienza”, P.le A. Moro 5, 00185 Rome, Italy
[2]Department of Industrial Engineering, University of Rome “Tor Vergata”, Via del Politecnico 1, 00133
[3]Department of Electronic Engineering, University of Rome “Tor Vergata”, Via del Politecnico 1, 00133
**mauro.tomassetti@uniroma1.it; *federico.marini@uniroma1.it*

[SM]

Numerical files of all experimental curves shown in Figures, 2 and 3, numerical file of discharging curves and numerical file of charging curves are placed in a compressed file zip named “Raw Fuel Cell charge and discharge data”.
